# Changes in tree community structure in defaunated forests are not driven only by dispersal limitation

**DOI:** 10.1002/ece3.6133

**Published:** 2020-03-09

**Authors:** Kirstie Hazelwood, C. E. Timothy Paine, Fernando H. Cornejo Valverde, Elizabeth G. Pringle, Harald Beck, John Terborgh

**Affiliations:** ^1^ Biological and Environmental Sciences University of Stirling Stirling UK; ^2^ Environmental and Rural Science University of New England Armidale NSW Australia; ^3^ Proyecto Castaña Madre de Dios Peru; ^4^ Department of Biology Program in Ecology, Evolution and Conservation Biology University of Nevada Reno NV USA; ^5^ Department of Biological Sciences Towson University Towson MD USA; ^6^ Department of Biology and Florida Museum of Natural History University of Florida Gainesville FL USA; ^7^ College of Science and Engineering James Cook University Cairns Qld Australia

**Keywords:** Amazon, defaunation, hunting, seed dispersal, tree recruitment

## Abstract

1. Bushmeat hunting has reduced population sizes of large frugivorous vertebrates throughout the tropics, thereby reducing the dispersal of seeds. This is believed to affect tree population dynamics, and therefore community composition, because the seed dispersal of large‐seeded trees depends upon large‐bodied vertebrates.
2. We report on a long‐running study of the effect of defaunation on a tropical tree community. In three censuses over 11 years, we compared sapling recruitment between a hunted and a nonhunted site, which are nearby and comparable to one another, to determine the extent to which species composition has changed through time following defaunation. We expected to find a reduced abundance of tree species that rely on large frugivores for dispersal at the hunted site and altered community structure as a consequence.
3. Although community composition at the hunted site diverged from that at the nonhunted site, the changes were independent of dispersal syndrome, with no trend toward a decline in species that are dispersed by large, hunted vertebrates. Moreover, the loss of large‐bodied dispersers did not generate the changes in tree community composition that we hypothesized. Some species presumed to rely on large‐bodied frugivores for dispersal are effectively recruiting despite the absence of their dispersers.
4. Synthesis: The presumption that forests depleted of large‐bodied dispersers will experience rapid, directional compositional change is not fully supported by our results. Altered species composition in the sapling layer at the hunted site, however, indicates that defaunation may be connected with changes to the tree community, but that the nature of these changes is not unidirectional as previously assumed. It remains difficult to predict how defaunation will affect tree community composition without a deeper understanding of the driving mechanisms at play.

## Graphical abstract

Our results indicate that forests suffering declines of large‐bodied dispersers do not experience rapid, directional compositional change. We do, however, detect altered species composition in the sapling layer at the hunted site, indicating that defaunation may be connected with changes to the tree community, but the nature of these changes is not unidirectional as previously assumed

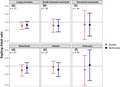

## 1 INTRODUCTION

Bushmeat hunting has caused population declines in many species of large vertebrates in forests throughout the tropics (Abernethy, Coad, Taylor, Lee, & Maisels, 2013; Dirzo et al., 2014; Peres & Palacios, 2007; Wright, Hernandéz, & Condit, 2007). This has given rise to a proliferation of defaunated “empty forests” (Redford, 1992), which, though they appear structurally intact, have suffered dramatic reductions in ecosystem function (Harrison et al., 2013; Peres, Thaise, Schietti, Desmoulieres, & Levi, 2015; Wright, Stoner, et al., 2007). Because animals interact with plant communities through herbivory, seed predation, pollination, and seed dispersal, altering these functions can alter the population dynamics of plants and have a cascading effect through the ecological community (Terborgh, 2013).

Hunting in Neotropical forests targets large‐bodied vertebrates, including many frugivores (Peres & Palacios, 2007), which play an essential role in seed dispersal of trees (Peres & van Roosmalen, 2002). Large‐seeded tree species are most likely to rely on large‐bodied frugivores for dispersal, because smaller animals are unable to swallow their seeds. Thus, any loss of large‐bodied frugivores is likely to impact dispersal function (Forget & Jansen, 2007). Undispersed seeds and the seedlings that germinate from them experience increased mortality because of high density‐dependent mortality close to the parent tree, which may have strong impacts on tree community structure (Bagchi et al., 2010; Comita, Muller‐Landau, Aguilar, & Hubbell, 2010; Swamy & Terborgh, 2010). We would therefore expect lower recruitment of species that experience reduced dispersal in hunted forests and, as a consequence, changes in plant community composition.

To date, there have been considerable inconsistencies in the results from studies that address the question of how tree communities are impacted by defaunation, with many suggesting that the loss of large‐bodied frugivores could have severe consequences for many tree species, including extinction (Nuñez‐Iturri, Olsson, & Howe, 2008; Peres et al., 2015; Terborgh et al., 2008), whereas others make more moderate assertions (Bagchi et al., 2018; Brocardo, Zipparro, Lima, Guevara, & Galetti, 2013; Kurten, Wright, & Carson, 2015). Numerous studies have assessed the effects on plant community composition in defaunated forests at a single point in time (Nuñez‐lturri & Howe, 2007; Peres et al., 2015), whereas others have conducted manipulative experiments over a longer time period on seeds or seedlings (Beck, Snodgrass, & Thebpanya, 2013; Brocardo et al., 2013; Kurten et al., 2015). Very few studies, however, have examined population dynamics in detail across ontogenetic stages over a period of more than 3 years, with the notable exception of Harrison et al. (2013), who found significant changes in tree community composition 15 years after the onset of hunting in south‐east Asia.

Studies that incorporate transitions between ontogenetic stages are important for predicting community dynamics. In a thorough evaluation of the effects of defaunation, Terborgh et al. (2008) compared the sapling and mature tree populations between nearby hunted and nonhunted forests in Peru, and concluded that defaunation had strongly impacted tree community composition. However, because their study was conducted at a single point in time, its ability to detect directional changes in composition was limited. Furthermore, their primary response metric for tree recruitment was the species‐specific ratio of saplings to adults at each site. As individual stems can remain in the sapling layer for decades (Connell & Green, 2000; Green, Harms, & Connell, 2014), many of the saplings they investigated could have derived from seeds that dispersed and germinated prior to the commencement of hunting (32 years previous to the study). In the current study, we build upon the work of Terborgh et al. (2008) with three census periods of the same hunted and nonhunted forests over 11 years, allowing finer temporal resolution to assess the effects of defaunation on tropical tree communities.

We hypothesized that defaunation would induce changes in sapling community structure and thus changes to the mature tree community in the long term. In particular, in hunted areas, we predicted declines in the abundances of tree species with large seeds and of tree species dispersed by large primates (Levey, 1987). To test these predictions, we first evaluate the comparability of the two sites, including comparisons of their mature tree community structure and light availability. We then examine whether there were differences in vertebrate abundance that could be attributed to differences in hunting pressure. Finally, we examine the degree to which species composition differs between sapling and mature tree communities, and whether these differences could be attributed to seed mass or dispersal syndrome. Our study uses trees from the sapling stage (>1 m height and <10 cm DBH [diameter at breast height]) to the mature tree stage (>10 cm DBH), and these stages were monitored three times over an 11‐year period. Using repeated observations designed to quantify the sapling recruits, which were likely to have germinated after the onset of hunting, we determined how the relative abundance of tree species has changed in a defaunated forest. We also assessed the potential for directional changes in community structure through time.

## 2 MATERIALS AND METHODS

### 2.1 Study site

The study took place at two sites in the Department of Madre de Dios, south‐east Peru. The intact site, which has not been subjected to hunting in the last 100 years, was near Cocha Cashu Biological Station (CCBS) in the core zone of Manu National Park (11°54′S, 71°22′W). The hunted site is near the settlement of Boca Manu in the buffer zone of the National Park (12°16′S, 70°54′W). The two sites, 100 km apart, are comparable, as they are both located in young alluvial soils of the Manu River floodplain, and neither has experienced commercial logging. Condit et al. (2002) indicated that tree communities under 100 km apart usually vary little throughout South America. We expected the tree communities at the two sites, therefore, to be similar in the absence of anthropogenic disturbance. The site near Boca Manu has been hunted since the establishment of the settlement in 1972, resulting in severely depleted communities of large mammals and birds (Terborgh et al., 2008). Because the mature trees present at the outset of the study probably recruited before hunting began (i.e., they had germinated more than 32 years previously), we expected the mature trees at both sites to be products of forests with fully intact disperser communities. In fact, the mature tree stands at the two sites have previously been shown to have similar species composition and abundance (Terborgh et al., 2008). In contrast, saplings currently recruiting at the hunted site should reflect the effects of defaunation.

### 2.2 Tree plots

We surveyed tree and sapling plots in 2004, 2009, and 2015 at the hunted site, and in 2006, 2010, and 2015 at the nonhunted site. Tree plots covered one 4‐ha plot at each site, with a central square 1‐ or 1.08‐ha sapling plot at the hunted and nonhunted sites, respectively. In each census, every individual was identified, tagged, and mapped, DBH was measured for trees (≥10 cm DBH) and large saplings (>1 cm DBH, <10 cm DBH), and height was measured for smaller saplings (>1 m height, <1 cm DBH). Over the three censuses at both sites, 94.6% of the individuals were identified to species level, 4.9% were identified to family or genus, and 0.5% of trees could not be identified. Vouchers were taken for individuals that were difficult to identify and later assigned final species classifications by experienced members of the field team.

We sought to maximize the probability that the saplings used in the study had germinated after hunting began. Because individuals can remain in the sapling stage for more than the 32 years since hunting began at our hunted site (Connell & Green, 2000; Green et al., 2014), we define “recruits” as individuals that recruited into the sapling layer (i.e., have reached 1 m in height) between the first and second or between the second and third censuses. These new recruits are most likely to be less than 32 years old compared to other saplings, and thus, it is likely that they germinated after the onset of hunting. Focussing on recruits, as opposed to on the whole sapling community, should more accurately represent the effect of hunting on the tree community.

### 2.3 Light

To assess the effects of light availability on sapling growth and recruitment, canopy cover was measured throughout the sapling plots using methods similar to those used in Welden, Hewett, Hubbell, and Foster (1991) and Terborgh et al. (2008). Parallel lines 5 m apart were walked across each sapling plot, starting 1 m into the plot to eliminate any effects of cutting along grid lines, with measurements taken every 5 m along the line. Each measurement assessed the presence or absence of canopy cover below 5 m (scoring 1), between 5 m and 20 m (scoring 2), and above 20 m (scoring 3). Scores were summed resulting in a score between 1 and 6 for each measurement. Light scores were compared between sites using Pearson's chi‐squared test of independence using R 3.3.0 (R Core Team, 2017).

### 2.4 Mammal surveys

Densities of vertebrate abundance were assessed at both sites in 2004 and 2015 using line transects, where all mammals and birds targeted by hunters were recorded. A transect was walked at each site 14 times during the dry season. Transects were 4 km in length on one trail, except for transects at the nonhunted site in 2015, which were 3 km. Surveys were carried out between 1 and 7 days apart. Transects incorporated a variety of vegetation types. Seven line transects were walked during the day and seven during the night at each site, between 28 June and 28 September. We used the methods of Peres (1999), walking at a speed of 1 km/hr during the day and the night in the dry season. Data collected for each mammal sighting included group size, species (or functional group if the species was unknown), and perpendicular distance from the transect line to the individual or to the center of the group. Data from 2004 were published in Terborgh et al. (2008), and data from 2015 are analyzed for a repeat comparison in this study.

For analysis, bird and mammal species were categorized into one of eight functional groups: large primates; small primates; nocturnal arboreal mammals, including night monkeys (*Aotus nigriceps*); large birds, including guans (*Penelope jacquacu*), curassows (*Mitu tuberosum*), and trumpeters (*Psophia leucoptera*); large terrestrial mammals, including jaguar (*Panthera onca*), armadillos (*Priodontes maximus, Dasypus novemcinctus*), deer (*Mazama americana*), tapir (*Tapirus terrestris*), and peccary (*Tayassu tajacu*); large rodents, including agouti (*Dasyprocta variegata*) and paca (*Cuniculus paca*); small nocturnal mammals, including rodents (*Proechimys* spp.), and opossums (*Marmosa* spp., *Didelphis* sp*.*, *Marmosops* spp.); and squirrels (*Sciurus* spp., *Sciurillus* spp., *Microsciurus* spp.). Bats were not counted in mammal transects because we do not expect their abundance to be impacted by hunters.

We used the Distance package in R 3.3.0 to compare vertebrate abundance between forests (Miller, 2015; R Core Team, 2017). Detection function models, in which the probability of detecting an animal decreases with distance from the transect line, were performed separately for each functional group. Detection functions and covariates differed among functional groups, with hazard rate functions used in rodent and large bird estimates. Species, group size, and time of transect (diurnal or nocturnal) were incorporated as covariates where model fit was improved with their addition. Chi‐square Cramer–von Mises goodness‐of‐fit tests assessed model fits. Models were selected on an AIC basis. Functional group densities were compared between sites using z‐tests. P values were obtained using Satterthwaite approximations for effective degrees of freedom.

### 2.5 Dispersal syndromes

Tree species were assigned to primary dispersal syndromes based on data collected over many years of observation at CCBS; 48% of classifications were derived from published data (Foster & Janson, 1985; Romo, 1996; Swamy et al., 2011; Terborgh, 1983), complemented by unpublished data and personal observations for 41% of classifications (11% of species). Where the dispersal syndrome was unknown, a species was assigned the same dispersal syndrome as other species in the same genus if there was a dominant dispersal syndrome for the genus. Tree species were assigned to one of six dispersal categories: abiotic: wind or autochorous, 11%; birds or bats, 32%; large primates (*Ateles* sp. or *Alouatta* sp. *Cebus* spp.), 22%; small arboreal mammals (small primates, squirrels, nocturnal arboreal mammals), 21%; terrestrial mammals (rodents, peccaries, tapirs), 3%; and unknown, 11%.

Heavier seeds are more likely to rely on large‐bodied dispersers because smaller dispersers are unable to handle or swallow them, so seed mass was assigned to each species. Seed mass was sourced from local data collected at CCBS (Pringle, Álvarez‐Loayza, & Terborgh, 2007; Terborgh, Álvarez‐loayza, Dexter, Cornejo, & Carrasco, 2011; Terborgh, Zhu, Álvarez‐Loayza, & Cornejo Valverde, 2014) and supplemented by published data collated in the Royal Botanic Gardens, Kew Seed Information Database (Royal Botanic Gardens Kew, 2017). Out of 641 tree species identified, 96% were assigned a seed mass. A seed mass was assigned to 37% of species at species level, 42% at genus level, and 17% at family level.

### 2.6 Data analyses

Analyses were carried out in R 3.3.0 (R Core Team, 2017) using the packages distance, vegan, smatr, pbkrtest, and lme4 (Bates, Maechler, Bolker, & Walker, 2015; Halekoh & Hojsgaard, 2014; Miller, 2015; Oksanen et al., 2017; Warton, Duursma, Falster, & Taskinen, 2012).

To assess the similarity of the hunted and nonhunted sites, we conducted three tests. First, we compared the relative abundance distribution of the mature trees between the two forests using major axis regression. Second, we evaluated the Bray–Curtis dissimilarity in species composition between sites. Third, we assessed the relative abundance of trees belonging to each seed dispersal syndrome between sites. In each test, high similarity would suggest that the dynamics of tree recruitment in the prehunted era at the hunted site were comparable to those of the nonhunted tree community.

To assess differences in sapling communities since the onset of hunting, we used Bray–Curtis dissimilarity to compare species composition at the two sites. Sapling communities were compared between (a) sites, (b) census years, and (c) growth stages, taking into account full sapling communities at each growth stage, and saplings that recruited at each of the latter two censuses (i.e., new sapling recruits) at each growth stage. Nonmetric multidimensional scaling (NMDS) ordinations were used to visualize the community structure. Compositional differences were tested for significance using a permutational multiple analysis of variance (PERMANOVA).

Linear mixed‐effect models were used to assess the causes of differences in species assemblage between the two sites. Dispersal syndrome category and seed mass were each allowed to interact with site (hunted or nonhunted) as fixed effects. Because the sapling layer at the hunted site may have been influenced by hunting, we used the sapling recruit to mature tree ratio (per species and within site) as the response variable to detect differences driven by hunting. We assigned random intercepts to species because overall abundance and sapling‐to‐mature tree ratios may vary among species for other reasons. Ratios were log‐transformed to reduce heteroscedasticity. The model was weighted by the population size of each species totaled over the two sites, with more common species having a stronger weight than rarer species. The significance of the interaction was tested through parametric bootstrapping, in which residuals and random effects are resampled to obtain estimated *F* values on which to base *p* values (Halekoh & Hojsgaard, 2014).

We excluded from all analyses species that did not occur as both mature trees and saplings, species occurring at a density of <1 mature tree·ha^−1^, and species that are known to mature at <10 cm DBH (final species pool: *N* = 97).

## 3 RESULTS

### 3.1 Site comparison

The hunted and nonhunted sites were similar with regard to the density, basal area, species richness, species composition, and representation of dispersal syndromes of mature trees (Table S1). The species composition of mature trees did not differ between sites at any census (permutational MANOVA of Bray–Curtis dissimilarity: *R* ^2^ = .08, *p* = .24). A major axis regression indicated that species abundance was similar at the two sites (*R* ^2^ = .42, *p* < .001), with a positive association between abundance within species at the two sites (Figure S1). The density of mature trees did not differ significantly between the two sites in any of the six dispersal categories (linear mixed‐effect model: *X* ^2^ = 22.99, *p* = .14; Figure S2). Comparison of canopy openness, as a proxy for light availability, showed there was higher light availability at the hunted site (*X* ^2^ = 10.24, *p* = .006). Terborgh et al. (2008) found no difference in canopy openness in 2004. A large treefall in the hunted plot between 2004 and 2009 is a likely cause of the difference in canopy openness between the two sites. Nevertheless, with respect to their mature tree communities, the hunted and nonhunted sites were highly comparable.

### 3.2 Mammal surveys

**Figure 1:**
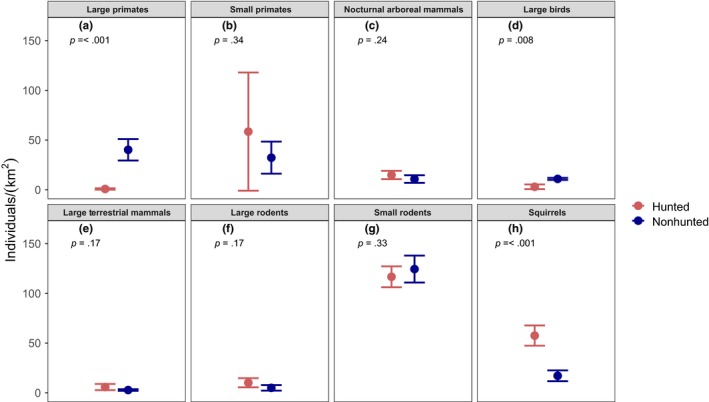
Density estimates for mammals and large birds divided into eight functional groups for hunted (red) and nonhunted (blue) sites. Error bars represent ±1 standard errors. The density of large primates was significantly reduced and the density of squirrels significantly increased, in the hunted compared to the nonhunted site

In 2015, a total of 182 km of transect were walked at the two sites, split equally between day and night, with 503 sightings recorded. Mammal and large bird population densities were estimated for the hunted and nonhunted site for eight functional groups (Figure 1). The population density of large primates at the hunted site was only 2% of that at the nonhunted site (0.82 and 40.23 individuals/km^2^, respectively; *Z* = −3.65, *p* < .001, Figure 1a), with only one group of large primates (*Ateles chamek*) consisting of three individuals recorded at the hunted site. Large bird population density was also significantly lower at the hunted site (*Z* = −3.05, *p* = .008, Figure 1d), consistent with Terborgh et al. (2008). Squirrel densities, on the other hand, were significantly higher at the hunted site (*Z* = −3.51, *p* = <.001. Figure 1h). Other groups of vertebrates did not vary in population density between sites, such as small rodents and opossums, which were most frequently observed, with >115 individuals/km^2^ at both sites.

### 3.3 Species compositional dissimilarity

**Figure 2:**
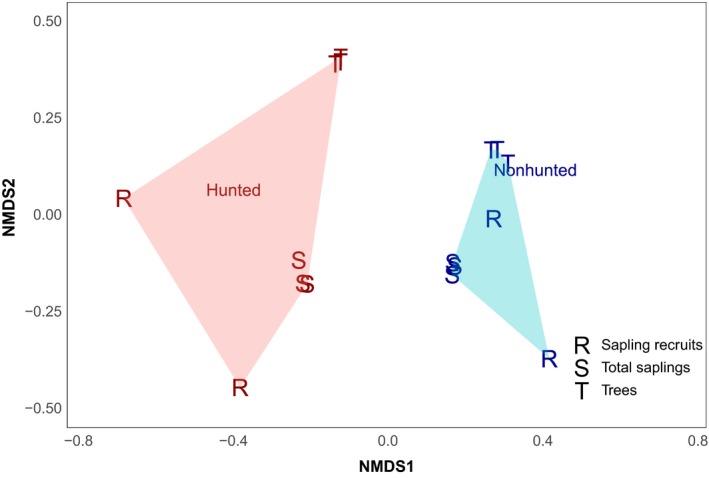
Nonmetric multidimensional scaling plot showing Bray–Curtis dissimilarities between sites (hunted: red, nonhunted: blue), censuses, and growth stages. dissimilarities between saplings and trees, and between recruits and trees were greater at the hunted site than at the nonhunted site. Points clustered together for Trees and Total Saplings are data points for each of the three censuses. Stress value is 0.03

At both sites, species composition differed between sapling and mature tree stages (*R* ^2^ = .78, *p* < .001, Figure 2). Dissimilarity between sapling recruits and mature trees was significantly greater at the hunted site than at the nonhunted site (*R* ^2^ = .96, *p* < .001). Thus, changes in species composition through time are occurring more rapidly at the hunted than at the nonhunted site. At the hunted site, dissimilarity between sapling recruits and mature trees increased from the second to the third census (Census 2:0.68, Census 3:0.76), whereas dissimilarity indices at the nonhunted site remained stable (Census 2:0.65, Census 3:0.63).

### 3.4 Dispersal syndromes

**Figure 3:**
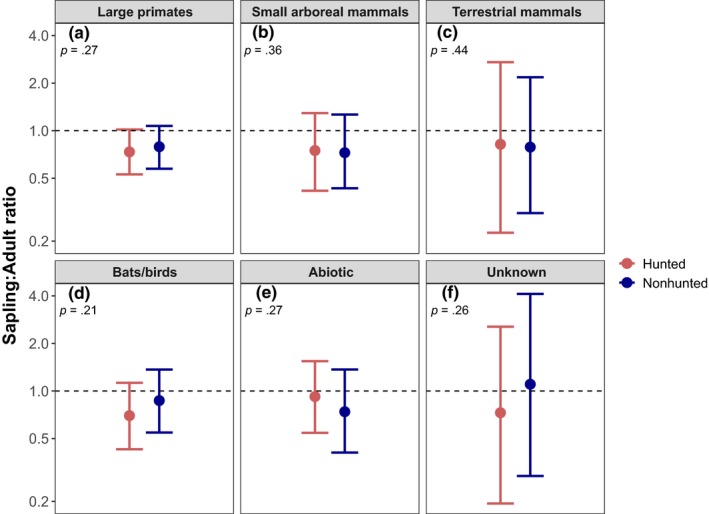
Sapling‐to‐adult density ratios, broken down by site and dispersal syndrome. Predicted values from a linear mixed‐effect model showing species‐specific log sapling‐to‐adult ratio by assigned dispersal mechanism for each site, with species as a random effect. Error bars represent ±1 standard errors. Model results show no significant difference in sapling‐adult ratio between two sites for any dispersal syndrome

**Figure 4:**
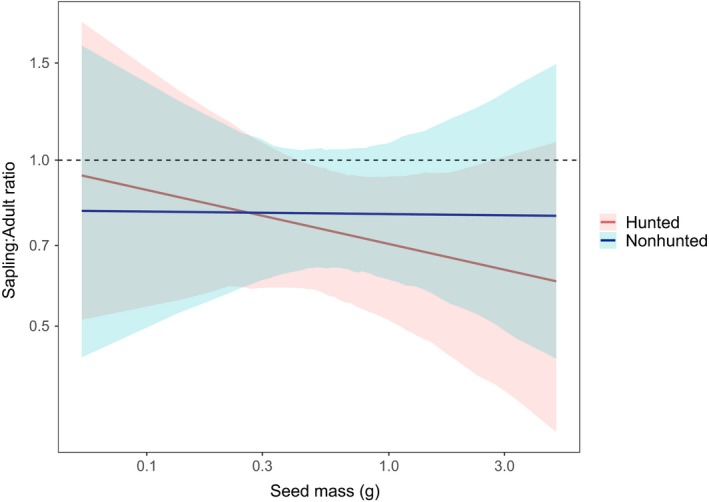
Predicted values from a linear mixed‐effect model showing species‐specific sapling‐to‐adult ratio as predicted by seed mass for each site, with species as a random effect. Model results show no significant difference in sapling‐to‐adult ratio where sites and seed mass are interacting effects (*p* = .26)

We examined sapling‐to‐adult density ratios from the 2009 and 2015 censuses to assess the degree to which defaunation‐induced changes in seed dispersal have generated changes in sapling recruitment. Although slightly more saplings recruited per adult for species dispersed by large primates at the nonhunted site, this difference was not significant (*SE* = 0.16, *p* = .27, Figure 3a). Moreover, dispersal syndrome as a whole did not predict differences in sapling‐to‐adult ratios between sites (*X* ^2^ = 12.53, *p* = .46). Seed mass also failed to predict sapling‐to‐adult ratio (*X* ^2^ = 3.39, *p* = .2, Figure 4); sapling‐to‐adult ratio predicted by interacting seed mass and site resulted in a marginally steeper slope at the hunted site than at the nonhunted site.

## 4 DISCUSSION

The results presented here are inconsistent with studies that have demonstrated reductions in the recruitment of large primate‐dispersed tree species following defaunation (Harrison et al., 2013; Kurten et al., 2015; Nuñez‐Iturri et al., 2008; Terborgh et al., 2008; Wright, Stoner, et al., 2007). Few of the studies addressing this question, however, have either examined various ontogenetic stages or extended over more than three years. For example, in a previous comparison of these sites conducted at a single point in time, the direction of compositional change following defaunation appeared to be affected by dispersal syndrome at the hunted site (Terborgh et al., 2008). Static data in this context, however, must be regarded with caution. Because trees can take many years to mature (Connell & Green, 2000; Green et al., 2014), studies performed at a single point in time can include saplings of uncertain age, including those that dispersed and germinated both before and after the onset of hunting. The repeated censuses in our study allowed us to assess sapling recruitment at five‐year intervals, and the period of 43 years since the onset of hunting gives us confidence that the community of recruiting saplings germinated in a hunted forest. Moreover, because the present findings contradict those of the previous study by Terborgh et al. (2008), the temporal extension of the study has had a profound impact on the interpretation of the detected patterns.

Although the mature tree communities were similar between sites (Table S1, Figures S1 and S2), the densities of key animal functional groups differed (Figure 1). In the 2015 census reported here, large primates showed lower densities at the hunted site, as did large birds to a smaller degree. These results are supported by previous studies carried out in Amazonia (Endo et al., 2010; Peres & Palacios, 2007; Terborgh et al., 2008), which also found reduced populations of large primates at hunted sites compared to nonhunted sites. These results confirm that hunting is severely depleting the fauna at the hunted site.

We hypothesized that the severe reduction in the abundance of large frugivorous dispersers would impact tree community structure by reducing the dispersal of tree species that rely on these extirpated fauna, thereby increasing the mortality of their seeds and seedlings. Our results confirmed that tree community structure is changing more rapidly in the hunted than in the nonhunted forest (Figure 2), consistent with the results of previous studies (Kurten et al., 2015; Nuñez‐Iturri et al., 2008). Notably, the saplings that recruited after the start of the study were more dissimilar from the mature trees at the hunted site than at the nonhunted site, and this dissimilarity increased between the second and third census in our study (Figure 2). We show, uniquely, increasing dissimilarity over time between the community of the newest saplings and that of the mature trees suggests not only that community structure will change in the next generation, but also that the rate of change is accelerating.

Nevertheless, and contrary to our prediction, neither dispersal syndrome nor seed mass explained the observed variation in sapling‐to‐mature tree ratios between hunted and nonhunted sites (Figures 3 and 4, Text S1). Like Bagchi et al. (2018), we found that the survival of large primate‐dispersed species is not uniformly limited by the loss of large primates. In fact, some tree species that rely on large primates for seed dispersal appeared to increase in abundance at the hunted site (e.g., *Theobroma cacao*): These species appear to be germinating and recruiting into the sapling layer in the absence of their presumed principal disperser. There are two potential explanations for these results: (a) Species thought to rely on extirpated fauna for dispersal are dispersing by alternative means or (b) such species are escaping mortality beneath the parent tree and surviving. These two scenarios are discussed below.

The persistence at the hunted site of some large primate‐dispersed species, while others declined in abundance, may reflect differences among species in secondary dispersal. Few studies have quantified the proportion of species that rely on secondary seed dispersers, though it is thought to play an essential role in the successful germination of many plant species (Andresen, 1999; Guimaraes, Galetti, & Jordano, 2008; Hirsch, Kays, Pereira, & Jansen, 2012). Species usually considered to play minor roles in seed dispersal, such as squirrels, which have a higher abundance at the hunted site, may opportunistically compensate for missing large primate dispersers (Bagchi et al., 2018). Or, if more undispersed seeds fall to the ground at the hunted site, then they may be dispersed by nonhunted rodents, including agoutis and squirrels (Hirsch et al., 2012). Indeed, pacas (*Cuniculus paca*) and agoutis (*Dasyprocta variegata*) are known to scatterhoard large seeds, and seed survival of scatterhoarded seeds is substantially higher than of those left on the surface (Galetti et al., 2010). Thus, scatterhoarding may favor survival of large seeds and effectively compensate germination and survival in the absence of a primary disperser. The extent to which these interactions can maintain a stable population for any plant species is unknown, but some studies addressing the continuing survival of plant species that rely on extinct mega‐fauna have suggested that compensatory dispersal by secondary dispersers can be long‐lasting and effective (Guimaraes et al., 2008). It is unlikely, however, that these compensatory mechanisms are effective for all species that are impacted by the loss of a primary disperser. Nor is it clear whether these mechanisms can maintain tree species at similar densities and distributions seen prior to defaunation. We have been surprised by some aspects of the results in this study, which is a clear indication that there is much yet to be learned about alternative pathways of seed dispersal in tropical forests.

Although the focus of this study has mainly been on the loss of frugivorous dispersers, hunting can also impact populations of seed predators (Galetti, Donatti, Pires, Guimarães, & Jordano, 2006; Wright et al., 2000). Simultaneous removal of dispersers and seed predators can have compensatory effects, when seeds that remain undispersed also remain un‐predated, potentially escaping density‐dependent mortality (Dirzo, Mendoza, & Ortiz, 2007; Kurten et al., 2015). This compensatory effect could also partly explain the lack of difference in large‐seeded tree species between the hunted and nonhunted site that we detected in this study, if large mammals are the main seed predators for undispersed large seeds. However, there is a large body of evidence that suggests that large mammals play a comparatively minor role in density‐dependent mortality of undispersed seeds when compared to pathogens or insects or small mammals (Beck et al., 2013; Notman & Villegas, 2005; Paine, Beck, & Terborgh, 2016). It seems unlikely therefore that any compensatory effects of reduced large mammal seed predators are strong enough to counteract the loss of large‐bodied dispersers.

Our detailed assessment of community dynamics across different ontogenetic stages allows insight into changes in tree community structure, but practical considerations limited us to two sites. Some caution should thus be exercised in extrapolating from these results. Although climatic conditions are consistently very similar between the two sites, microhabitats comprising variation in soil nutrients, light, and humidity may vary. For example, although multiple smaller treefalls occurred at both sites, a large treefall at the hunted site may have favored fast‐growing, light‐dependent, and abiotically dispersed species. Nevertheless, there is no reason to think that these circumstances should have favored primate‐dispersed or large‐seeded species, and thus, these treefalls should not have impacted the lack of a relationship between dispersal syndrome or seed size and forest type. Moreover, we note that the adult tree communities were very similar between the sites (Table S1), which demonstrates that whatever the microhabitat differences between our sites may be, similarity in tree community structure is at least possible.

Better predictions of population dynamics can be made for species that have short‐lived seed banks or rapid growth during early ontogenetic stages. Slow‐growing species and those with long‐lived seed banks take longer to experience changes in their population densities, and such changes could remain undetected even at the timescale of our study. It is unlikely, however, that a substantial proportion of the plant community set seed more than forty years ago and still have not reached 1 m in height (Connell & Green, 2000; Green et al., 2014). Thus, we remain confident that the majority of the saplings recruiting into this community have been subject to the effects of defaunation. These difficulties render long‐term studies such as this one essential to the understanding of changing ecological processes in natural systems that are becoming rapidly more susceptible to defaunation and other human‐induced impacts (Abernethy et al., 2013; Dirzo et al., 2014; Peres, 2000).

This is the first time that a community assemblage in a hunted forest has been observed over such an extended time period (11 years), assessing tree communities a full 43 years after the onset of hunting. Our utilization of new sapling recruits—distinct from the full sapling community—is unique to this study. This approach allowed us to eliminate the potential confounding effects of recruitment time lags that may be present in statically sampled sapling communities of unknown age. This combination of an extended period of study and a better knowledge of the age of recruiting saplings means we can be confident that saplings in the cohorts of new recruits were dispersed after the onset of hunting.

This study suggests that complex and species‐specific mechanisms are driving changes in tree community structure following defaunation. These results suggest that the outcome for plant communities where hunting is taking place will not be completely clear‐cut, with declines in all large‐seeded or large mammal‐dispersed species. Instead, it appears that tree communities will change in as yet unpredicted ways and that understanding these changes will require more detailed insights into ecological processes and species interactions. Our study also suggests that previous studies may have overestimated the impacts of defaunation by assuming uniform declines in the recruitment of large‐seeded species following the loss of large primates (Peres et al., 2015). Predictions about long‐term changes to tropical tree communities and the cascading effects that arise from defaunation will be inaccurate if impacts are assumed to be identical across all species, and we hope that further investigations into the processes associated with disperser loss will shed some light on what directional changes we should expect in a defaunated landscape.

## CONFLICT OF INTEREST

None declared.

## AUTHORS CONTRIBUTIONS

JT conceived the ideas, JT and HB designed the methodology, all authors collected the data, KH and CETP analyzed the data, KH, CETP, and JT led the writing of the manuscript. All authors contributed critically to the drafts and gave final approval for publication.

## Data Availability

Data are archived in DRYAD data storage at https://doi.org/10.5061/dryad.wpzgmsbhg.
