# Supplementary figures and images for: Changes in tree community structure in defaunated forests are not driven only by dispersal limitation
Source: Ecol Evol. 2020 Mar 9;10(7):3392–401. doi: 10.1002/ece3.6133 (PMC7140993; doi:10.1002/ece3.6133)

Figure S1

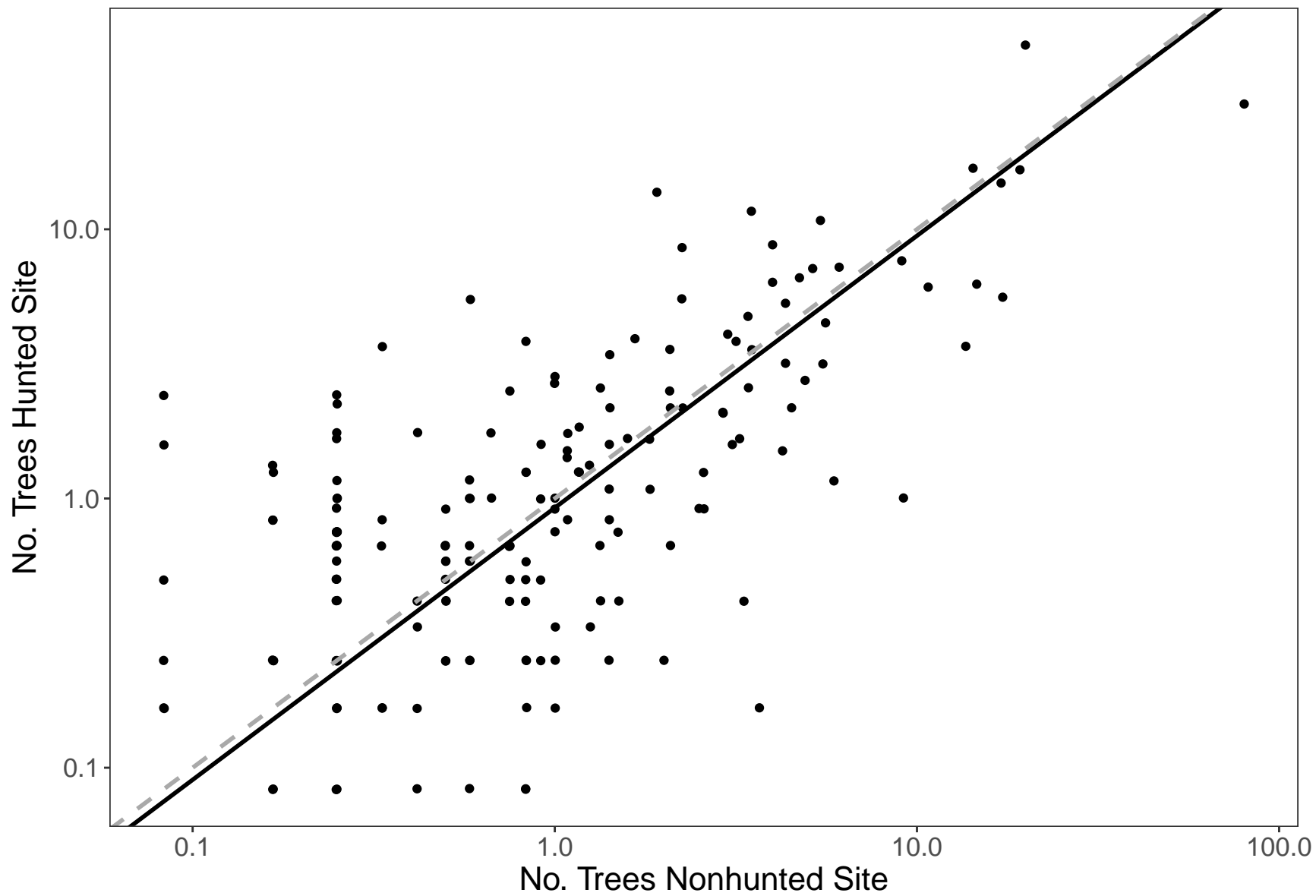

Supplement: Supplementary file 1 [file ECE3-10-3392-s001.pdf]

Figure S2

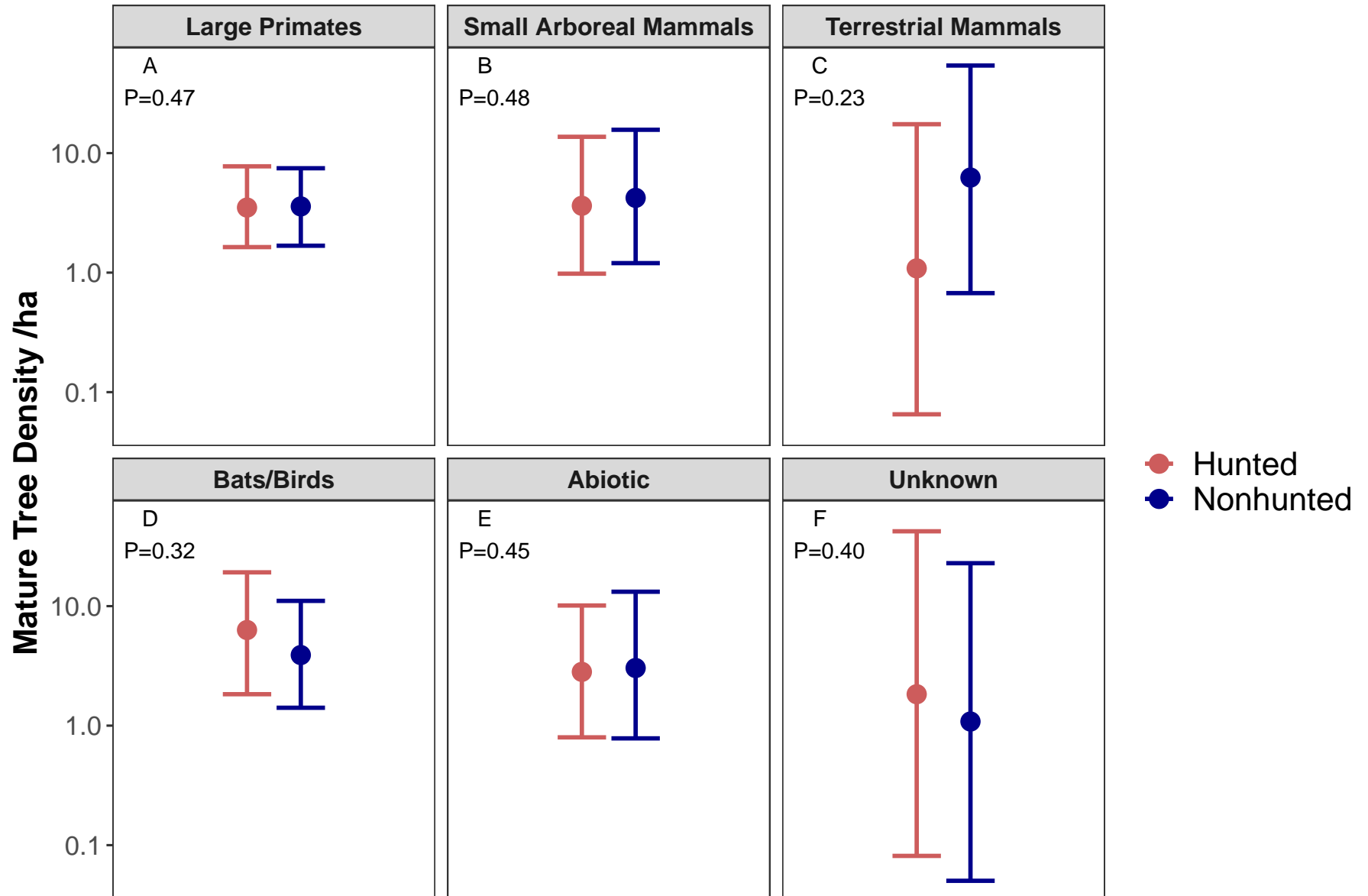

Supplement: Supplementary file 2 [file ECE3-10-3392-s002.pdf]

Figure S3

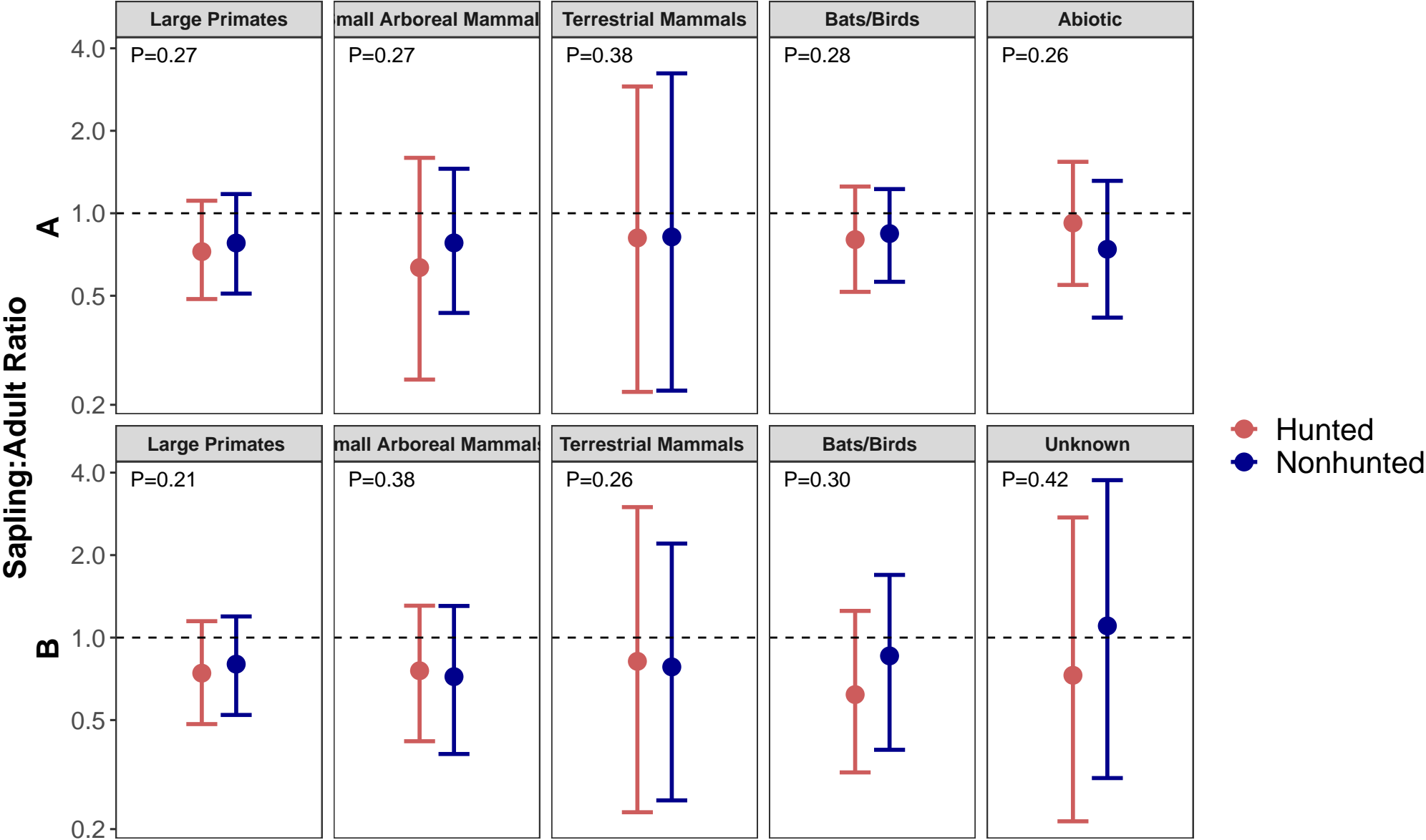

Supplement: Supplementary file 3 [file ECE3-10-3392-s003.pdf]
