# Supplementary material for: Changes in tree community structure in defaunated forests are not driven only by dispersal limitation
Source: Ecol Evol. 2020 Mar 9;10(7):3392–401. doi: 10.1002/ece3.6133 (PMC7140993; doi:10.1002/ece3.6133)
Supplement: Supplementary file 4 [file ECE3-10-3392-s004.docx]

**Supplementary Materials**

**Tables**

Table S1: Comparison of mature adult stands between hunted and nonhunted sites and comparison of sapling recruits and mature trees within site at all three census’s. Bray-Curtis dissimilarities are bounded between 0 and 1.

|  | Census 1 | | Census 2 | | Census 3 | |
| --- | --- | --- | --- | --- | --- | --- |
|  | Hunted | Nonhunted | Hunted | Nonhunted | Hunted | Nonhunted |
| Mature Tree Density pha | 1912 | 1900 | 1945 | 1967 | 1944 | 2023 |
| Mature Tree Basal Area (m^2^) | 115.5 | 108.5 | 114.2 | 102.9 | 114.8 | 111.9 |
| Species Richness | 225 | 256 | 234 | 262 | 244 | 271 |
| Bray-Curtis Dissimilarity of mature tree communities between sites | 0.39 | | 0.39 | | 0.4 | |

**Figures**

**Figure S1:** Comparison of adult tree abundance between hunted and non-hunted sites by species. Solid line represents major axis (MA) regression fit, dashed line shows regression where slope = 1 and intercept = 0. Axes are log transformed. Species abundances are similar at the two sites.

**Figure S2:** Linear mixed effect model predictions showing mature-tree density per hectare by dispersal syndrome at hunted (red) and non-hunted (blue) sites. Results show no significant difference between dispersal syndrome distribution between the two sites, indicating strong similarities between the mature tree communities.

**Figure S3:** Predicted values from linear mixed effect model showing species-specific log sapling-to-adult ratio by assigned dispersal mechanism for each site, with species as a random effect. Species are divided into (A) those that have only one named disperser and (B) those for which the named disperser is the primary but not the only disperser. Model results show no significant difference between the two sites within any dispersal syndrome.

**Text S1:** Results based on a single assigned disperser (Figure 3) could face some bias due to the coarse groupings of dispersal syndromes, when many species experience compensatory effects from secondary dispersal. To account for this we also assigned dispersal syndromes separately for species that rely on one disperser alone and for those that rely on a disperser among one or more other dispersers. When modelled against sapling-to-adult ratio using a linear mixed effect model there was still no significant effect of dispersal syndrome, (X^2^=18.72, p=0.57**).** Even when a species is reliant solely on large primates for dispersal, the reduced abundance of dispersers at the hunted site did not appear to be impacting recruitment (Figure S3-a).
